# Histamine and Immune Biomarkers in CNS Disorders

**DOI:** 10.1155/2016/1924603

**Published:** 2016-04-13

**Authors:** Ramón Cacabelos, Clara Torrellas, Lucía Fernández-Novoa, Francisco López-Muñoz

**Affiliations:** ^1^Chair of Genomic Medicine, Camilo José Cela University, C/Castillo de Alarcón 49, Villanueva de la Cañada, 28692 Madrid, Spain; ^2^EuroEspes Biomedical Research Center, Institute for CNS Disorders and Genomic Medicine, Santa Marta de Babio, Bergondo, 15165 Corunna, Spain; ^3^Neuropsychopharmacology Unit, Hospital 12 de Octubre Research Institute (i+12), Avenida de Córdoba, s/n, 28041 Madrid, Spain

## Abstract

Neuroimmune dysregulation is a common phenomenon in different forms of central nervous system (CNS) disorders. Cross-links between central and peripheral immune mechanisms appear to be disrupted as reflected by a series of immune markers (CD3, CD4, CD7, HLA-DR, CD25, CD28, and CD56) which show variability in brain disorders such as anxiety, depression, psychosis, stroke, Alzheimer's disease, Parkinson's disease, attention-deficit hyperactivity disorder, migraine, epilepsy, vascular dementia, mental retardation, cerebrovascular encephalopathy, multiple sclerosis, brain tumors, cranial nerve neuropathies, mental retardation, and posttraumatic brain injury. Histamine (HA) is a pleiotropic monoamine involved in several neurophysiological functions, neuroimmune regulation, and CNS pathogenesis. Changes in brain HA show an age- and sex-related pattern, and alterations in brain HA levels are present in different CNS regions of patients with Alzheimer's disease (AD). Brain HA in neuronal and nonneuronal compartments plays a dual role (neurotrophic versus neurotoxic) in a tissue-specific manner. Pathogenic mechanisms associated with neuroimmune dysregulation in AD involve HA, interleukin-1*β*, and TNF-*α*, whose aberrant expression contributes to neuroinflammation as an aggravating factor for neurodegeneration and premature neuronal death.

## 1. Introduction

In 1955, Pepys postulated that histamine (HA) could affect the immune response. Since then, the influence of HA on immune and inflammatory reactions has been well documented. In the late 1970s and early 1980s, Watanabe's, Schwartz's, and Panula's groups in Japan, France, and Finland, respectively, demonstrated the presence of HA in the brain where this ancestral biogenic amine acts as a neurotransmitter and neuroimmune modulator [1–3]. HA is at a privileged position to display multiple pleiotropic functions in peripheral tissues and in the central nervous system (CNS). HA is synthesized by histidine decarboxylase (HDC; EC 4.1.1.22) from L-histidine in different cellular compartments (mast cells, basophils, glial cells, endothelial cells, and neurons). HA acts through different types of HA receptors (H1R, H2R, H3R, and H4R). H1R and H2R are widely distributed in most cells and tissues; however, H3Rs are mainly expressed in the CNS, and H4Rs are expressed in hematopoietic cells, indicating their function in neurotransmission and immunomodulation, respectively. The H3R is a recognized drug target for neuronal diseases, such as cognitive impairment, schizophrenia (SCZ), sleep/wake disorders, epilepsy, and neuropathic pain [4]. The organization of the brain histaminergic system shows a strategic disposition, with HA neurons located in the posterior hypothalamus, from where ascending pathways (to the anterior hypothalamus, limbic structures (hippocampus), neocortex, and subcortical structures) and descending pathways (to the brain stem and spinal cord) are organized. The interplay of brain HA in neurons, endothelial cells, glia, and mast cells is fundamental for the regulation of diverse physiological functions (neuroendocrine system, circadian rhythms, sleep-wakefulness cycle, psychomotor activity, mood, learning, cognition, appetite, and eating behavior); and alterations in multicompartment brain HA are involved in several pathological conditions, such as epilepsy, stroke, anxiety, depression, psychosis, neurodegeneration, and neuroinflammatory processes [3, 5, 6].

## 2. Compartmentalization and Physiological Functions

Brain HA is distributed in several compartments (neuronal, glial, endothelial, and mast cells). Mast cells are connective tissue cells, discovered by Ehrlich in 1887, which are rich in metachromatic granules containing HA, heparin, and serotonin. Riley, West, and coworkers were the first to establish an association between mast cells and HA in 1953, and Benditt and colleagues showed, in 1955, that mast cells also contained 5-hydroxytryptamine (5-HT; serotonin). In 1953, Riley demonstrated that injection of HA releasers was followed by damage to mast cells. Examples of HA releasers include compound 48/80, mast cell-degranulating peptide (MCD), polylysine, polymyxin B, dextran, phosphatidylserine, melittin, F-Met-tripeptides, C5a/C3a anaphylatoxins, C4a, calcium ionophore A23187, and many cytokines and growth factors. Many, if not all, mast cells are innervated by varicose axons in different body structures, and even an “axon reflex” pathway involving mast cells was proposed in the skin by Kiernan in 1965 [7]. Mast cells originate from bone marrow hematopoietic CD34^+^ progenitor cells, which enter the circulation and migrate to distal tissues where they ultimately reside. Mature mast cells are a heterogeneous population of cells with various phenotypes depending on their location. Mast cells express Fc*ε*RI, a high-affinity IgE receptor, which upon activation induces exocytosis of cytoplasmic secretory granules,* de novo* synthesis of lipid-derived mediators, and release of cytokines, chemokines, monoamines, and growth factors. Mast cell secretory granules contain a great variety of mediators, such as histamine, serotonin, tryptase, chymase, and antizyme inhibitor 2 (AZIN2), an activator of polyamine biosynthesis whose catalyzing enzyme is ornithine decarboxylase [8]. Mast cells are resident in the brain and contain numerous mediators, including neurotransmitters, cytokines, and chemokines, that are released in response to a variety of natural and pharmacological triggers. Brain mast cells are of two types, namely, metachromatic type I cells and normochromatic type II cells, or neurolipomastocytoid cells. In mice devoid of mast cells (W/W^v^ mice), the administration of *α*-fluoromethylhistidine (FMH), a suicide inhibitor of HDC, reduces the concentration of brain HA to nearly zero, suggesting that approximately 50% of the content of HA in the CNS belongs to the nonneuronal pool [9, 10]. The presence of mast cells in meninges and perivascular locations on the brain side of the BBB, especially in thalamic and hippocampal regions, may indicate that this cell type is relevant in neurovascular responses. The number of mast cells in the brain fluctuates with stress and various behavioral and endocrine states. Some mast cell mediators are synthesized upon activation (e.g., neuropeptides, cytokines) while others are preformed and stored in granules from which they are rapidly released (e.g., serotonin, histamine). Mast cells can act via autocrine and paracrine mechanisms; their secretions can reach a large space volume, and they can migrate to critical regions influencing neuronal activity, repairing tissue damage, or aggravating inflammatory processes within the CNS. These properties suggest that mast cells are poised to influence neural systems underlying behavior. Using genetic and pharmacological loss-of-function models, Nautiyal et al. [11] performed a behavioral screen for arousal responses including emotionality, locomotor, and sensory components and found that mast cell-deficient Kit^W-sh/W-sh^ (sash^−/−^) mice had a greater anxiety-like phenotype than WT and heterozygote littermate control animals in the open field arena and elevated plus maze. Blockade of brain, but not peripheral, mast cell activation increased anxiety-like behavior. Brain mast cells might be implicated in the modulation of anxiety-like behavior and provide evidence for the behavioral importance of neuroimmune links [11].

Microglia can constitutively express HA receptors (H1R, H2R, H3R, and H4R), and the expression of H1R and H4R can be selectively upregulated by HA in microglial cells in a dose-dependent manner. HA can also stimulate microglia activation and production of proinflammatory cytokines. HA induces TNF-*α* and IL-6 release from activated microglia via H1R and H4R-MAPK and the PI3K/AKT-NF-kappa B signaling pathway [12]. Microglia express H2R, H3R, histidine decarboxylase, and histamine N-methyltransferase. Both forskolin-induced cAMP accumulation and ATP-induced intracellular Ca^2+^ transients are reduced by the H3R agonist imetit but not by the H2R agonist amthamine. H3Rs can regulate various microglial functions. HA and imetit inhibit microglial chemotaxis, phagocytosis, and lipopolysaccharide (LPS)-induced cytokine production [13]. HA and substance P can trigger microglial activation and release of proinflammatory factors from microglia, thus contributing to the development of microglia-mediated inflammation in the brain [14].

The interplay of brain HA in neurons, endothelial cells, glia, and mast cells is fundamental for the regulation of diverse physiological functions (neuroendocrine system, circadian rhythms, sleep-wakefulness cycle, psychomotor activity, mood, learning, cognition, appetite, and eating behavior); and alterations in multicompartment brain HA are involved in several pathological conditions [3, 5, 15, 16].

HA is implicated in the control of arousal state, exerting a potent phase-shifting effect on the circadian clock in the suprachiasmatic nucleus (SCN). To reset the circadian clock, HA increases [Ca^2+^]_i_ in SCN neurons by activating CaV 1.3 channels through H1Rs and secondarily by causing Ca^2+^-induced Ca^2+^ release from RyR-mediated internal stores [17]. Light has direct effects on sleep and wakefulness causing arousal in diurnal animals and sleep in nocturnal animals. Histaminergic neurotransmission attenuates the light-induced sleep response during the dark period [18]. Using knockout (KO) mice lacking HDC, Parmentier et al. [19] demonstrated the importance of histaminergic neurons in maintaining wakefulness under behavioral challenges. H1-receptor KO (H1^−/−^) mice share several characteristics with HDC KO mice, including a decrease in wakefulness after lights-off despite its normal baseline daily amount, a decreased EEG slow wave sleep (SWS)/W power ratio, and inability to maintain wakefulness in response to behavioral challenges. Most of these effects are mediated by central H1Rs [19]. Yu et al. [20] reported that GABA and HA are involved in the mechanisms of wakefulness. HA neurons in the tuberomammillary nucleus (TMN) of the hypothalamus form a widely projecting, wake-active network that sustains arousal. HA neurons contain GABA. Selective siRNA knockdown of the vesicular GABA transporter (vgat, SLC32A1) in HA neurons produces hyperactive mice with an exceptional amount of sustained wakefulness. Ablation of the vgat gene throughout the TMN further sharpens this phenotype. Optogenetic stimulation in the caudate-putamen and neocortex of HA axonal projections from the TMN evokes tonic, extrasynaptic GABAA receptor Cl^−^ currents onto medium spiny neurons and pyramidal neurons. These currents are abolished following vgat gene removal from the TMN area. Thus, wake-active HA neurons may generate a paracrine GABAergic signal that serves to provide a brake on overactivation of HA and to increase the precision of neocortical processing. Yu et al. [21] studied the contribution of one putative local clock in mouse histaminergic neurons in the tuberomammillary nucleus to the regulation of the sleep-wake cycle. Histaminergic neurons are silent during sleep and start firing after wake onset. HA enhances wakefulness. HDC gene expression varies with time of day. Deletion of the Bmal1 (Arntl/Mop3) clock gene from HA cells removes this variation, producing higher HDC expression and brain HA levels during the day. The consequences include more fragmented sleep, prolonged wakefulness at night, shallower sleep depth (lower nonrapid eye movement [NREM] *δ* power), increased NREM-to-REM transitions, hindered recovery sleep after sleep deprivation, and impaired memory. Removing BMAL1 from histaminergic neurons does not, however, affect circadian rhythms.

**Figure 1:**
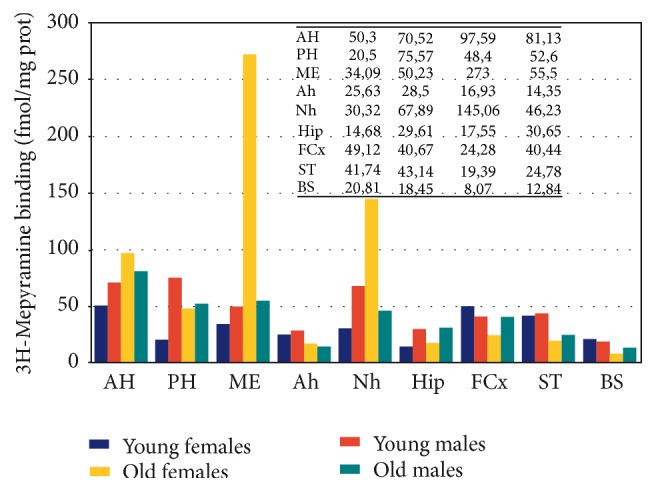
Age- and sex-related changes in brain histamine H1 receptors. AH: Anterior Hypothalamus; PH: Posterior Hypothalamus; ME: Median Eminence; Ah: Adenohypophysis; Nh: Neurohypophysis; Hip: Hippocampus; FCx: Frontal Cortex; ST: Striatum; BS: Brain Stem. Young rats < 3 months. Old rats > 12 months of age.

There are important age- and sex-related changes in the concentration of HA and H1R in different regions of the CNS [6, 22] (Figure 1). Dramatic changes in HA and H1R have been reported after surgical manipulation of the neuroendocrine system (e.g., castration, adrenalectomy), clearly indicating the relevant effect that changes in the hypothalamus-pituitary-gonadal axis and hypothalamus-pituitary-adrenergic axis exert on brain HA [9, 22]. Similarly, direct interactions between the somatotropinergic system (GRH/SS-GH-IGF1) and HA [23] and the vasopressinergic system and HA have been extensively demonstrated [24].

HA is a powerful regulator of neuroimmune function [7]. L-Histidine and HA induce a time- and dose-dependent decrease in hypothalamic interleukin-1*β* (IL-1*β*), and this effect is not affected by mepyramine (H1R antagonist), famotidine (H2R antagonist), or thioperamide (H3R antagonist) [25]. The abolishment of neuronal HA by *α*-fluoromethylhistidine, a suicide inhibitor of HDC, causes a significant decrease in the hypothalamic concentration of TNF-*α* [26].

## 3. Brain Disorders and Neurodegeneration

**Figure 2:**
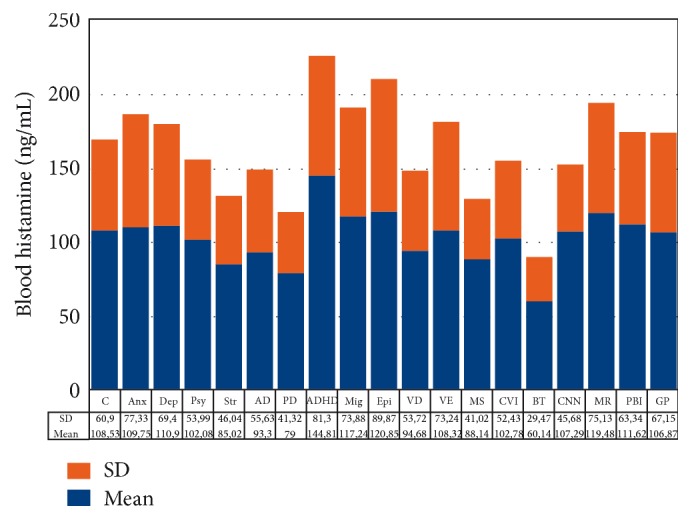
Blood histamine levels in CNS disorders. C: control; Anx: anxiety; Dep: depression; Psy: psychosis; Str: stroke; AD: Alzheimer's disease; PD: Parkinson's disease; ADHD: attention-deficit hyperactivity disorder; Mig: migraine; Epi: epilepsy; VD: vascular dementia; VE: vascular encephalopathy; MS: multiple sclerosis; CVI: cerebrovascular insufficiency; BT: brain tumors; CNN: cranial nerve neuropathy; MR: mental retardation; PBI: posttraumatic brain injury; GP: general population.

**Figure 3:**
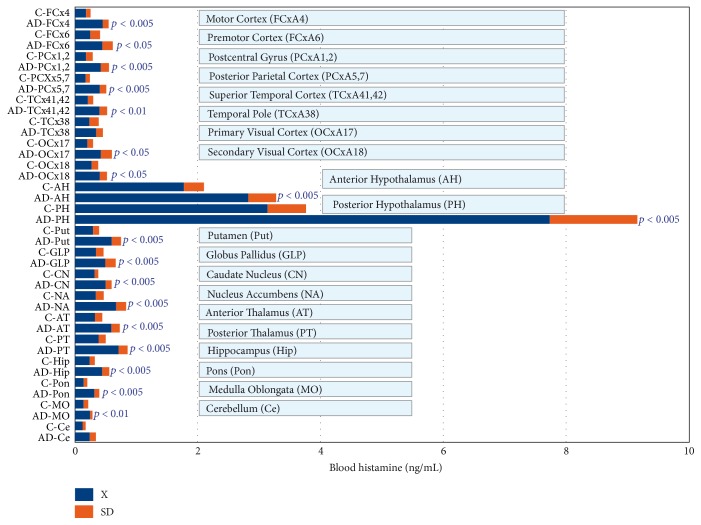
Changes in histamine levels in different brain regions from patients with Alzheimer's disease (AD). Notice the presence of a high concentration of HA in most brain regions of AD patients as compared with that of control (C) brains.

Important changes in the peripheral (Figure 2) and central levels of HA (Figure 3) occur in patients with different CNS disorders [6]. These changes are particularly important in Alzheimer's disease (AD), where HA levels are significantly increased in most CNS regions [6, 27] (Figure 3). HA, TNF-*α*, and IL-1*β* regulate each other in the hypothalamus. Changes in HA, TNF-*α* [28], and IL-1*β* [28, 29] in AD correlate with cerebrovascular dysfunction and cognitive decline [3, 30]. Furthermore, improvement in cognitive function with neuroprotectants (e.g., CDL-choline) [31–33] or vegetal neurotrophins (e.g., anapsos) [34, 35] can reverse alterations in HA, TNF-*α*, and IL-1*β* levels in AD and in animal models of dementia [7, 25, 34–37].

**Figure 4:**
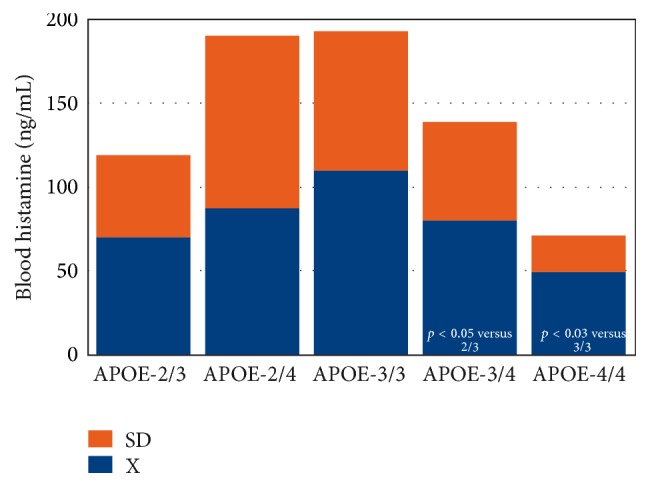
*APOE*-related blood histamine levels in Alzheimer's disease. Concentration of blood histamine in patients with Alzheimer's disease (AD) according to their apolipoprotein-E (*APOE*) genotype.* APOE* distribution and frequencies:* APOE*-2/3: 4%;* APOE*-2/4: 2%;* APOE*-3/3: 49%;* APOE*-3/4: 35%;* APOE*-4/4: 9%.

**Figure 5:**
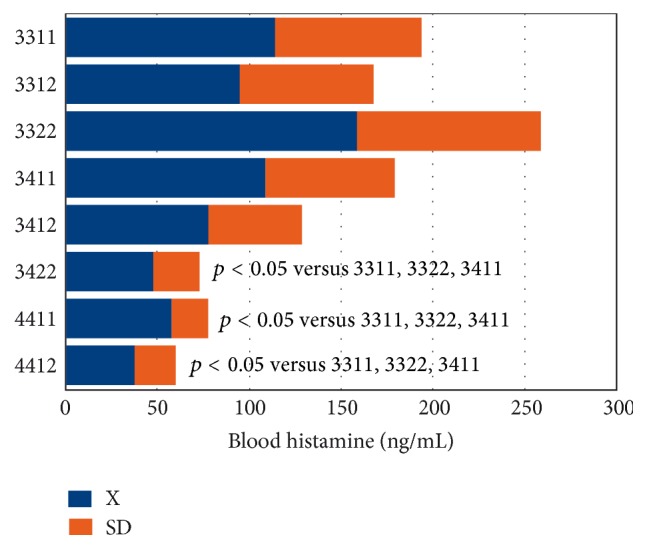
Bigenic genotype (*APOE* +* PSEN1*)-related histamine levels in Alzheimer's disease. Concentration of blood histamine in patients with Alzheimer's disease (AD) according to their bigenic genotypes integrating* APOE* + Presenilin-1 (*PSEN1*) in a single haplotype. Haplotype frequencies: 3311 (*APOE*-3/3 +* PSEN1*-1/1): 16%; 3312 (*APOE*-3/3 +* PSEN1*-1/2): 27%; 3322 (*APOE*-3/3 +* PSEN1*-2/2): 10%; 3411 (*APOE*-3/3 +* PSEN1*-1/1): 12%; 3412 (*APOE*-3/4 +* PSEN1*-1/2): 20%; 3422 (*APOE*-3/4 +* PSEN1*-2/2): 5.5%; 4411 (*APOE*-4/4 +* PSEN1*-1/1): 5.5%; 4412 (*APOE*-4/4 +* PSEN1*-1/2): 4%.

AD-related neurodegeneration exhibits a pathological phenotype compatible with a reactive neuroinflammatory process in which HA, TNF-*α*, and IL-1*β*, among many other immune effectors, are involved. There is also a correlation between microglia activation and* APOE* genotypes [38, 39]; and peripheral HA levels are also* APOE*-related, with the lowest levels present in patients harboring the* APOE*-4 allele [6, 40] (Figures 4 and 5). An association between* APOE*-4 and increased expression of CD95 on T cells has also been reported, suggesting that hyperexpression of Fas mRNA and surface Fas receptor on CD45RO^+^ T lymphocytes may explain the occurrence of inflammatory cellular infiltrates in AD brain tissue [41].

All these data together illustrate the potential role of HA as a fundamental player in AD-related neuroinflammation and other neuropsychiatric disorders [16]. In fact, results of clinical trials with H3 antagonists such as ABT-288 [42] or GSK239512 have been recently reported [43].

## 4. Peripheral Immune Markers in CNS Disorders

Patients with different types of CNS disorders exhibit significant variation in lymphocyte subsets with regard to that of the general population, indicating that alterations in brain function may alter lymphocytes and immune markers in the periphery. By flow cytometry analysis, we have studied changes in lymphocyte subpopulations and immune markers in healthy controls (*N* = 374) and in the general population (*N* = 3311), as well as in patients with diverse CNS disorders including anxiety (*N* = 340), depression (*N* = 483), psychosis/schizophrenia (*N* = 199), stroke (*N* = 77), Alzheimer's disease (*N* = 247), Parkinson's disease (*N* = 81), attention-deficit hyperactivity disorder (ADHD) (*N* = 47), migraine (*N* = 238), epilepsy (*N* = 81), vascular dementia (*N* = 215), vascular encephalopathy (associated with diabetes, hypertension, or symptomatic arteriosclerosis) (*N* = 404), multiple sclerosis (*N* = 22), chronic cerebral insufficiency (*N* = 152), brain tumors (*N* = 14), cranial nerve neuropathy (trigeminal neuralgia, facial palsy) (*N* = 32), mental retardation (*N* = 135), and posttraumatic brain injury (*N* = 65) [6] (Table 1). CD3 (total T cells) only showed significant differences (*p* < 0.05) in children with mental retardation and in patients with vascular dementia. CD4 (T helper/inducer cells) significantly differ between controls and ADHD, mental retardation, and epilepsy; between depression and ADHD, mental retardation, and epilepsy; between migraine and ADHD and mental retardation; and between cerebrovascular insufficiency, ADHD, and mental retardation. CD7 (T cells + Natural Killer, NK) differ between vascular encephalopathy and psychosis, migraine, epilepsy, and mental retardation. CD8 (T suppressor/cytotoxic) did not show any relevant difference among different CNS pathologies with regard to controls. HLA-DR (Class II MCH antigen) exhibit significant differences between ADHD and Parkinson's disease, stroke, vascular dementia, AD, and vascular encephalopathy; between epilepsy and Parkinson's disease, stroke, vascular dementia, AD, and vascular encephalopathy; and between mental retardation and Parkinson's disease, stroke, vascular dementia, AD, and vascular encephalopathy. CD25 (IL-2 receptors) were found lower in ADHD, mental retardation, and epilepsy with respect to controls and vascular encephalopathy. CD28 (60–80% CD3^+^) were found lower in vascular dementia and Parkinson's disease with regard to controls, anxiety, depression, psychosis, migraine, epilepsy, multiple sclerosis, and posttraumatic brain injury. CD56 (NK cells) were significantly increased in Parkinson's disease and vascular dementia in relation to most other groups. No significant changes were found in both CD3^+^/HLA-DR^+^ and CD4/CD8 ratios in CNS disorders [6] (Table 1). All these data together clearly reflect that brain damage and/or pathologic alterations in brain function leading to neurologic and/or psychiatric disorders can induce significant changes in peripheral lymphocytes and immune markers. This fact provides additional support to the predominant role of brain on immune function and immune mediators [6].

## 5. Inflammation Signaling and Regulation of Proinflammatory Genes

Recent studies bring new insights into the nature of inflammation signaling and regulation of proinflammatory genes, opening new avenues for novel therapeutic intervention in inflammatory processes. One example is the A20 protein, a cytosolic peptide that controls interactions between key ubiquitin-conjugating enzymes, thereby regulating the expression of proinflammatory genes [44, 45]. The* A20* gene locus has been associated with risk for Crohn's disease, systemic lupus erythematosus, rheumatoid arthritis, type I diabetes, psoriasis, and atherosclerosis.* A20* is an important regulator of autoimmunity and a tumor suppressor for Hodgkin's lymphoma and large B cell lymphoma.* A20* acts through the transcription factor nuclear factor-*κ*B (NF-*κ*B) to control inflammation, and NF-*κ*B regulates the expression of genes involved in inflammation and immunity. NF-*κ*B protein inhibitor (I*κ*B) maintains inactive NF-*κ*B in the cytoplasma of unstimulated cells. Proinflammatory ligands, such as TNF-*α*, LPS, and IL-1*β*, activate the I*κ*B kinase (IKK) complex to phosphorylate I*κ*Bs, inducing their degradation, which releases associated NF-*κ*B to translocate into the nucleus and activate the transcription of genes that promote inflammation. One target gene is* A20* (*TNFAIP3*), a negative feedback regulator that terminates IKK and NF-*κ*B activation [44]. Shembade et al. [45] have reported that A20 negatively regulates inflammation by inhibiting NF-*κ*B transcription factor in the TNF receptor (TNFR) and Toll-like receptor (TLR) pathways. A20 contains deubiquitinase and E3 ligase domains and has been proposed to function as a ubiquitin-editing enzyme downstream of TNFR1 by inactivating RIP1. A20 inhibits the E3 ligase activities of TRAF6, TRAF2, and clAP1 by antagonizing interactions with the E2 ubiquitin-conjugating enzymes Ubc13 and UbcH5c. A20, together with the regulatory molecule TAX1BP1, interacts with Ubc13 and UbcH5c and triggers their ubiquitination and proteasome-dependent degradation. These results suggest that pharmacological inhibition of the E2 ligases controlled by A20 might be beneficial in treating certain autoimmune diseases and cancer.

Another example is the role of mitochondria in inflammation. Injury causes a systemic inflammatory response syndrome (SIRS) that is clinically similar to sepsis. Microbial pathogen-associated molecular patterns activate innate immunocytes through pattern recognition receptors. Cellular injury can release endogenous damage-associated molecular patterns (DAMPs) that activate innate immunity. As postulated by Sagan, Altman, and others in the 1960s, mitochondria are evolutionary endosymbionts derived from bacteria which might bear bacterial molecular motifs. Zhang et al. [46] have shown that injury releases mitochondrial DAMPs (MTDs) into the circulation with functionally important immune consequences. MTDs include formyl peptides and mtDNA that activate polymorphonuclear neutrophils (PMNs) through formyl peptide receptor-1 and Toll-like receptor (TLR) 9, respectively. MTDs promote PMN calcium flux and phosphorylation of mitogen-activated protein (MAP) kinases, thus leading to PMN migration and degranulation. Circulating MTDs can elicit neutrophil-mediated organ injury. The release of such mitochondrial factors by cellular injury is a key link between trauma, inflammation, and SIRS [46].

Metabolic reprogramming is implicated in macrophage activation. The NOTCH1 pathway dictates activation of M1 phenotypes in isolated mouse hepatic macrophages (HMacs) and in a murine macrophage cell line by coupling transcriptional upregulation of* M1* genes with metabolic upregulation of mitochondrial oxidative phosphorylation and ROS (mtROS) to augment induction of* M1* genes. Enhanced mitochondrial glucose oxidation is achieved by increased recruitment of the NOTCH1 intracellular domain (NICD1) to nuclear and mitochondrial genes that encode respiratory chain components and by NOTCH-dependent induction of pyruvate dehydrogenase phosphatase 1 (Pdp1) expression, pyruvate dehydrogenase activity, and glucose flux to the TCA cycle. Inhibition of the NOTCH pathway or Pdp1 knockdown abrogates glucose oxidation, mtROS, and M1 gene expression. Conditional NOTCH1 deficiency in the myeloid lineage attenuates HMac M1 activation and inflammation in a murine model of alcoholic steatohepatitis and markedly reduces lethality following endotoxin-mediated fulminant hepatitis in mice. Monocyte tracking requires NOTCH1 for the migration of blood monocytes into the liver and subsequent M1 differentiation. According to the elegant studies reported by Xu et al. [47], NOTCH1 promotes reprogramming of mitochondrial metabolism for M1 macrophage activation.

Yin et al. [48] demonstrated for the first time that early hyperlipidemia promotes endothelial cells (EC) activation before monocyte recruitment via a caspase-1-sirtuin 1-activator protein-1 pathway, which provides an important insight into the development of novel therapeutics for blocking caspase-1 activation as early intervention of metabolic cardiovascular diseases and inflammations. Using new apolipoprotein E (*ApoE*)^−/−^/caspase-1^−/−^ double knockout mice, they observed that (i) early hyperlipidemia induced caspase-1 activation in* ApoE* ^−/−^ mouse aorta; (ii) caspase-1^−/−^/*ApoE* ^−/−^ mice attenuated early atherosclerosis; (iii) caspase-1^−/−^/*ApoE* ^−/−^ mice had decreased aortic expression of proinflammatory cytokines and attenuated aortic monocyte recruitment; and (iv) caspase-1^−/−^/*ApoE* ^−/−^ mice had decreased EC activation, including reduced adhesion molecule expression and cytokine secretion. Oxidized lipids activated caspase-1 and promoted pyroptosis in EC by a reactive oxygen species mechanism. Caspase-1 inhibition resulted in accumulation of sirtuin 1 in the* ApoE* ^−/−^ aorta, and sirtuin 1 inhibited caspase-1 upregulated genes via activator protein-1 pathway.

Tristetraprolin (TTP) is an anti-inflammatory protein that acts by binding to AREs in its target mRNAs, such as TNF mRNA, and promoting their deadenylation and decay. TNF released from inflammatory cells can then stimulate gene expression in tissue cells, such as fibroblasts. The decay rates of transcripts encoded by several early-response genes, including* Cxcl1*,* Cxcl2*,* Ier3*,* Ptgs2*, and* Lif*, are significantly slowed in TTP-deficient fibroblasts after TNF stimulation. These changes are associated with TTP-dependent increases in CXCL1, CXCL2, and IER3 protein levels. The TTP-susceptible transcripts contain multiple, conserved, closely spaced, potential TTP binding sites in their 3′-UTRs. WT TTP, but not a nonbinding TTP zinc finger mutant, binds to RNA probes that are based on the mRNA sequences of* Cxcl1*,* Cxcl2*,* Ptgs2*, and* Lif*. TTP-promoted decay of transcripts encoding chemokines and other proinflammatory mediators is thus a critical posttranscriptional regulatory mechanism in the response of secondary cells, such as fibroblasts, to TNF released from primary immune cells [49].

Neutrophils have the capacity to package a variety of cytokines into cytoplasmic granules for subsequent release upon inflammatory conditions. The rapid secretion of cytokines orchestrates the action of other immune cells at the infection site and contributes to the development and chronicity of inflammatory diseases. Naegelen et al. [50] studied the intracellular SNARE machinery responsible for the regulation of cytokine secretion and degranulation and demonstrated that syntaxin-3 (STX3) is required for the maximal release of IL-1*α*, IL-1*β*, IL-12b, and CCL4 without alteration of other cytokine secretions in dHL-60 cells. STX3 is involved in MMP-9 exocytosis from gelatinase granules, where STX3 is partly localized. The secretion of IL-1*α*, IL-1*β*, IL-12b, and CCL4 occurs during gelatinase degranulation, a process controlled by STX3 which has an essential role in trafficking pathways of cytokines in neutrophil granulocytes.

Sestrins (Sesns) are conserved antioxidant proteins that accumulate in cells in response to various stresses. Yang et al. [51] investigated whether Sesn2 regulates Toll-like receptor- (TLR-) mediated inflammatory signaling and sought to identify the molecular mechanism responsible. Sesn2 almost completely inhibits LPS-induced NO release and iNOS expression. A gene knockdown experiment confirmed the role of Sesn2 in LPS-activated RAW264.7 cells. Proinflammatory cytokine release and expression are inhibited in Sesn2-expressing cells. Sesn2 prevents LPS-elicited cell death and ROS production via inhibition of NADPH oxidase. NF-*κ*B and AP-1 are redox-sensitive transcription factors that regulate the expressions of diverse inflammatory genes. Sesn2 specifically inhibits AP-1 luciferase activity and its DNA binding, but not those of NF-*κ*B. AP-1 inhibition by Sesn2 is due to a lack of JNK, p38, and c-Jun phosphorylation. Sesn2 protects galactosamine (Gal)/LPS-induced liver injury in mice infected with a recombinant adenovirus Sesn2 (Ad-Sesn2). Ad-Sesn2 causes less severe hepatic injury as supported by decreases in the ALT, AST, and hepatocyte degeneration. Ad-Sesn2 attenuates Gal/LPS-induced proinflammatory gene expression. Sesn2 inhibits TLR-induced proinflammatory signaling and protects cells by inhibiting JNK- or p38-mediated c-Jun phosphorylation.

## 6. Conclusions

Abundant data provide support to a multidimensional crosstalk among neural, endocrine, and immune signals probably contributing to global homeostasis, constant surveillance to maintain control against exogenous and/or endogenous stressors, brain maturation and development, neuroprotection, and fine-tuning of brain functions associated with higher activities of the CNS. Neuroimmune dysfunction may be part of the pathogenesis of different CNS disorders, including mental disorders (SCZ, depression, anxiety, and posttraumatic stress disorder), neurodegenerative disorders (AD, Parkinson's disease, and Prion disease), brain infections, stroke, brain tumors, and demyelinating disorders. It is likely that the neuroimmune system acts as a regulatory, defense system which reacts against noxious stimuli that endanger brain function stability and optimum performance. In this context, neuroimmune activation in CNS pathology might be a double-faced reactive phenomenon: (i) neuroprotective, when the stimulus activating the neuroimmune cascade is able to neutralize tissue damage and/or brain dysfunction and (ii) neurotoxic, when the reactive neuroinflammatory event is persistent and becomes an autoaggression which magnifies the damage. This Sword of Damocles (dual role) hanging over the CNS and other peripheral tissues under central control must be exquisitely balanced to avoid dangerous decompensation (dominance of neurotoxic effects over neuroprotective effects). This homeostatic equilibrium can be maintained by the interplay of different cytokines, chemokines, neurotransmitters, neuropeptides, neurohormones, and pleiotropic substances such as histamine. This biogenic amine, acting on different receptors or emanating from different sources (neuronal histamine versus nonneuronal histamine) can exert antagonistic effects (neurotrophic versus neurotoxic) on neuronal targets inducing either protection or damage. Although there was a growing interest for neuroimmune function and neuroinflammation during the past two decades, with relevant scientific contributions to the field, our impression is that the present knowledge on neuroimmune mechanisms and their pathological consequences is still at a very primitive stage. Therapeutic intervention to halt progression of deleterious neuroinflammatory reactions in CNS disorders is a major challenge for molecular psychoneuropharmacology in the future [6].

## Figures and Tables

**Table 1:** Immune markers in CNS disorders.

Population	*N*	Age (years)	CD3 (%)	CD4 (%)	CD7 (%)	CD8 (%)	HLA-DR (%)	CD25 (%)	CD28 (%)	CD56 (%)	CD3^+^/HLA-DR^+^	CD4/CD8
Control	374	49.87 ± 15.30^(1–3)^	71.11 ± 8.08^(95-96)^	51.45 ± 8.35^(97–99)^	65.57 ± 11.57	27.51 ± 7.44	12.31 ± 4.02	19.96 ± 9.26^(126–128)^	58.72 ± 9.88^(131-132)^	15.24 ± 6.42	6.21 ± 3.97	2.06 ± 0.85
Anxiety	340	42.69 ± 14.46^(4–6)^	69.73 ± 7.82	49.83 ± 7.77	63.41 ± 14.51	26.33 ± 7.13	12.58 ± 4.92	17.61 ± 10.06	58.20 ± 10.56^(133-134)^	15.15 ± 7.04	4.94 ± 3.80	2.06 ± 0.79
Depression	483	43.19 ± 14.11^(7–9)^	70.30 ± 7.98	50.92 ± 7.97^(100–102)^	63.80 ± 13.79	25.92 ± 7.26	12.32 ± 4.89	17.96 ± 9.94	58.97 ± 10.99^(135-136)^	14.56 ± 7.26	5.04 ± 4.44	2.16 ± 0.89
Psychosis	199	32.42 ± 13.19^(10-11)^	69.90 ± 7.47	48.32 ± 7.52	66.20 ± 12.50^(107)^	27.75 ± 7.88	12.93 ± 4.47	17.62 ± 9.08	59.44 ± 10.16^(137-138)^	14.40 ± 7.84	5.36 ± 4.72	1.92 ± 0.77
Stroke	77	61.51 ± 13.36^(12–20)^	70.57 ± 7.52	49.93 ± 8.11	61.57 ± 12.50	27.67 ± 7.69	10.25 ± 3.91	17.69 ± 8.98	54.32 ± 10.44	18.67 ± 8.31	6.30 ± 5.00	1.98 ± 0.73
Alzheimer's disease	247	70.10 ± 11.11^(21–33)^	67.61 ± 10.34	49.60 ± 9.18	60.62 ± 14.36	26.24 ± 9.81	11.72 ± 6.20	17.65 ± 9.21	52.43 ± 10.89	18.92 ± 8.88	6.58 ± 4.85	2.25 ± 1.19
Parkinson's disease	81	67.82 ± 10.03^(34–46)^	66.94 ± 10.63	47.54 ± 9.52	62.12 ± 13.65	28.22 ± 10.24	10.30 ± 3.88	15.64 ± 8.16	50.41 ± 9.97	22.44 ± 8.55^(149–162)^	5.95 ± 3.39	2.00 ± 1.06
Attention-Deficit Activity Disorder	47	9.27 ± 3.54	68.31 ± 6.45	44.29 ± 5.92	66.46 ± 10.32	25.90 ± 5.75	15.53 ± 5.10^(111–115)^	9.80 ± 5.74	56.82 ± 11.09	11.70 ± 5.22	4.12 ± 2.80	1.79 ± 0.46
Migraine	238	38.77 ± 13.90^(47-48)^	70.19 ± 8.56	50.17 ± 8.62^(103-104)^	66.15 ± 11.31^(108)^	27.25 ± 7.23	12.30 ± 4.19	17.11 ± 9.25	60.06 ± 10.50^(139-140)^	15.45 ± 8.15	5.43 ± 4.54	2.01 ± 0.84
Epilepsy	81	30.81 ± 19.06^(49-50)^	67.94 ± 7.18	46.36 ± 9.17	66.22 ± 12.77^(109)^	28.55 ± 7.08	14.17 ± 5.22^(116–120)^	15.07 ± 6.86	57.76 ± 9.97^(141-142)^	16.26 ± 7.59	5.25 ± 3.72	1.75 ± 0.65
Vascular dementia	215	75.29 ± 7.51^(51–65)^	66.48 ± 10.54	48.37 ± 9.60	61.05 ± 14.46	27.27 ± 8.45	11.85 ± 6.27	17.55 ± 9.53	51.05 ± 10.91	20.40 ± 8.15^(163–167)^	6.71 ± 5.44	2.00 ± 1.00
Vascular encephalopathy	404	68.78 ± 11.88^(66–78)^	68.35 ± 8.49	49.31 ± 9.11	59.86 ± 14.25	27.25 ± 8.88	12.07 ± 5.71	19.10 ± 10.23^(129-130)^	52.71 ± 11.22	18.08 ± 8.00	6.53 ± 4.73	2.10 ± 1.09
Multiple sclerosis	22	38.72 ± 11.64^(79-80)^	70.68 ± 7.81	49.42 ± 8.73	61.84 ± 17.94	26.57 ± 6.71	13.10 ± 5.73	17.94 ± 8.87	60.84 ± 9.02^(143-144)^	13.05 ± 7.07	5.47 ± 3.30	2.06 ± 1.06
Cerebrovascular insufficiency	152	53.64 ± 16.54^(81–83)^	68.88 ± 9.74	49.61 ± 9.22^(105-106)^	62.80 ± 12.29	27.80 ± 8.97	12.58 ± 6.21	18.35 ± 9.96	56.70 ± 12.12	16.57 ± 7.60	6.65 ± 6.12	2.03 ± 0.94
Brain tumors	14	54.21 ± 16.68^(84–86)^	68.66 ± 8.17	48.00 ± 8.66	65.16 ± 11.09	27.41 ± 9.50	11.75 ± 3.86	15.58 ± 6.31	53.08 ± 12.85	18.83 ± 7.19	5.25 ± 3.33	2.01 ± 1.01
Cranial nerve neuropathy	32	48.78 ± 18.61^(87–89)^	69.58 ± 7.10	48.82 ± 10.69	61.06 ± 13.07	24.79 ± 9.65	12.31 ± 5.25	15.41 ± 9.42	54.34 ± 15.30	15.31 ± 8.86	6.24 ± 5.13	2.21 ± 0.80
Mental retardation	135	14.63 ± 11.91	67.05 ± 7.95	45.95 ± 7.83	68.38 ± 10.38^(110)^	25.92 ± 5.70	15.69 ± 5.44^(121–125)^	12.02 ± 7.46	56.68 ± 11.75^(145-146)^	14.00 ± 6.50	5.72 ± 3.96	1.86 ± 0.62
Posttraumatic brain injury	65	39.43 ± 16.99^(90-91)^	70.86 ± 7.53	49.39 ± 9.32	64.48 ± 13.96	28.08 ± 7.42	12.10 ± 4.41	15.85 ± 10.02	59.25 ± 8.74^(147-148)^	15.87 ± 7.61	5.89 ± 4.14	1.93 ± 0.79
General population	3311	49.89 ± 21.44^(92–94)^	69.15 ± 8.84	49.32 ± 8.89	63.50 ± 13.36	27.15 ± 8.17	12.46 ± 5.33	17.66 ± 9.67	56.15 ± 11.23	16.53 ± 7.94	5.99 ± 4.85	2.04 ± 0.93
		*p* < 0.001^*∗*^	*p* < 0.001	*p* < 0.001	*p* < 0.001	*p* = 0.039	*p* < 0.001	*p* < 0.001	*p* < 0.001	*p* < 0.001	*p* < 0.001	*p* = 0.002

*Values*: mean ± standard deviation.

Control (C), anxiety (Anx), depression (Dep), psychosis (Psy), stroke (Str), Alzheimer's disease (AD), Parkinson's disease (PD), attention-deficit hyperactivity disorder (ADHD), migraine (Mig), epilepsy (Epi), vascular dementia (VD), vascular encephalopathy (VE), multiple sclerosis (MS), cerebrovascular insufficiency (CVI), brain tumors (BT), cranial nerve neuropathy (CNN), mental retardation (MR), posttraumatic brain injury (PBI), and general population (GP).

^*∗*^Kruskal-Wallis One Way ANOVA on Ranks

Dunn's Method (*p* < 0.05): (1) C versus ADHD; (2) C versus MR; (3) C versus Psy; (4) Anx versus ADHD; (5) C versus MR; (6) Anx versus Psy; (7) Dep versus ADHD; (8) Dep versus MR; (9) Dep versus Psy; (10) Psy versus ADHD; (11) Psy versus MR; Str versus MR; (12) Str versus ADHD; (13) Str versus MR; (14) Str versus Psy; (15) Str versus Epi; (16) Str versus MS; (17) Str versus Mig; (18) Str versus PBI; (19) Str versus Anx; (20) Str versus Dep; (21) AD versus ADHD; (22) AD versus MR; (23) AD versus Psy; (24) AD versus Epi; (25) AD versus MS; (26) AD versus Mig; (27) AD versus PBI; (28) AD versus Anx; (29) AD versus Dep; (30) AD versus CNN; (31) AD versus C; (32) AD versus GP; (33) AD versus CVI; (34) PD versus ADHD; (35) PD versus MR; (36) PD versus Psy; (37) PD versus Epi; (38) PD versus MS; (39) PD versus Mig; (40) PD versus PBI; (41) PD versus Anx; (42) PD versus Dep; (43) PD versus CNN; (44) PD versus C; (45) PD versus GP; (46) PD versus CVI; (47) Mig versus ADHD; (48) Mig versus MR; (49) Epi versus ADHD; (50) Epi versus MR; (51) VD versus ADHD; (52) VD versus MR; (53) VD versus Psy; (54) VD versus Epi; (55) VD versus MS; (56) VD versus Mig; (57) VD versus PBI; (58) VD versus Anx; (59) VD versus Dep; (60) VD versus CNN; (61) VD versus C; (62) VD versus GP; (63) VD versus CVI; (64) VD versus BT; (65) VD versus Str; (66) VE versus ADHD; (67) VE versus MR; (68) VE versus Psy; (69) VE versus Epi; (70) VE versus MS; (71) VE versus Mig; (72) VE versus PBI; (73) VE versus Anx; (74) VE versus Dep; (75) VE versus CNN; (76) VE versus C; (77) VE versus GP; (78) VE versus CVI; (79) MS versus ADHD; (80) MS versus MR; (81) CVI versus ADHD; (82) CVI versus MR; (83) CVI versus Psy; (84) BT versus ADHD; (85) BT versus MR; (86) BT versus Psy; (87) CNN versus ADHD; (88) CNN versus MR; (89) CNN versus Psy; (90) PBI versus ADHD; (91) PBI versus MR; (92) GP versus ADHD; (93) GP versus MR; (94) GP versus Psy; (95) C-CD3 versus MR-CD3; (96) C-CD3 versus VD-CD3; (97) C-CD4 versus ADHA-CD4; (98) C-CD4 versus MR-CD4; (99) C-CD4 versus Epi-CD4; (100) Dep-CD4 versus ADHA-CD4; (101) Dep-CD4 versus MR-CD4; (102) Dep-CD4 versus Epi-CD4; (103) Mig-CD4 versus ADHD-CD4; (104) Mig-CD4 versus MR-CD4; (105) CVI-CD4 versus ADHD-CD4; (106) CVI-CD4 versus MR-CD4; (107) Psy-CD7 versus VE-CD7; (108) Mig-CD7 versus VE-CD7; (109) Epi-CD7 versus VE-CD7; (110) MR-CD7 versus VE-CD7; (111) ADHD-HLA-DR versus PD-HLA-DR; (112) ADHD-HLA-DR versus Str-HLA-DR; (113) ADHD-HLA-DR versus VD-HLA-DR; (114) ADHD-HLA-DR versus AD-HLA-DR; (115) ADHD-HLA-DR versus VE-HLA-DR; (116) Epi-HLA-DR versus PD-HLA-DR; (117) Epi-HLA-DR versus Str-HLA-DR; (118) Epi-HLA-DR versus VD-HLA-DR; (119) Epi-HLA-DR versus AD-HLA-DR; (120) Epi-HLA-DR versus VE-HLA-DR; (121) MR-HLA-DR versus PD-HLA-DR; (122) MR-HLA-DR versus Str-HLA-DR; (123) MR-HLA-DR versus VD-HLA-DR; (124) MR-HLA-DR versus AD-HLA-DR; (125) MR-HLA-DR versus VE-HLA-DR; (126) C-CD25 versus ADHD-CD25; (127) C-CD25 versus MR-CD25; (128) C-CD25 versus Epi-CD25; (129) VE-CD25 versus ADHD-CD25; (130) VE-CD25 versus MR-CD25; (131) C-CD28 versus PD-CD28; (132) C-CD28 versus VD-CD28; (133) Anx-CD28 versus PD-CD28; (134) Anx-CD28 versus VD-CD28; (135) Dep-CD28 versus PD-CD28; (136) Dep-CD28 versus VD-CD28; (137) Psy-CD28 versus PD-CD28; (138) Psy-CD28 versus VD-CD28; (139) Mig-CD28 versus PD-CD28; (140) Mig-CD28 versus VD-CD28; (141) Epi-CD28 versus PD-CD28; (142) Epi-CD28 versus VD-CD28; (143) MS-CD28 versus PD-CD28; (144) MS-CD28 versus VD-CD28; (145) MR-CD28 versus PD-CD28; (146) MR-CD28 versus VD-CD28; (147) PBI-CD28 versus PD-CD28; (148) PBI-CD28 versus VD-CD28; (149) PD-CD56 versus ADHD-CD56; (150) PD-CD56 versus MS-CD56; (151) PD-CD56 versus MR-CD56; (152) PD-CD56 versus Psy-CD56; (153) PD-CD56 versus Dep-CD56; (154) PD-CD56 versus CNN-CD56; (155) PD-CD56 versus Mig-CD56; (156) PD-CD56 versus Anx-CD56; (157) PD-CD56 versus C-CD56; (158) PD-CD56 versus PBI-CD56; (159) PD-CD56 versus Epi-CD56; (160) PD-CD56 versus GP-CD56; (161) PD-CD56 versus CVI-CD56; (162) PD-CD56 versus VE-CD56; (163) VD-CD56 versus ADHD-CD56; (164) VD-CD56 versus MS-CD56; (165) VD-CD56 versus MR-CD56; (166) VD-CD56 versus Psy-CD56; (167) VD-CD56 versus Dep-CD56.

## References

[1] Watanabe T., Wada H. (1991). *Histaminergic Neuros: Morphology and Function*.

[2] Cacabelos R., Watanabe T., Wada H. (1990). Histaminergic regulation of the neuroendocrine system. *Histaminergic Neurons: Morphology and Functions*.

[3] Fernández-Novoa L., Cacabelos R. (2001). Histamine function in brain disorders. *Behavioural Brain Research*.

[4] Tiligada E., Zampeli E., Sander K., Stark H. (2009). Histamine H3 and H4 receptors as novel drug targets. *Expert Opinion on Investigational Drugs*.

[5] Baronio D., Gonchoroski T., Castro K., Zanatta G., Gottfried C., Riesgo R. (2014). Histaminergic system in brain disorders: lessons from the translational approach and future perspectives. *Annals of General Psychiatry*.

[6] Cacabelos R., Torrellas C., Fernández-Novoa L., Aliev G. (2016). Neuroimmune crosstalk in CNS disorders: the histamine connection. *Current Pharmaceutical Design*.

[7] Cacabelos R., Fernández-Novoa L., Franco-Maside A., Álvarez X. A. (1992). Neuroimmune function of brain histamine: implications for neurotrophic activity and neurotoxicity. *Annals of Psychiatry*.

[8] Kanerva K., Lappalainen J., Mäkitie L. T., Virolainen S., Kovanen P. T., Andersson L. C. (2009). Expression of antizyme inhibitor 2 in mast cells and role of polyamines as selective regulators of serotonin secretion. *PLoS ONE*.

[9] Cacabelos R., Yamatodani A., Fukui H. (1985). Nature of histaminergic neuromodulation of the corticotropinergic system. *Biogenic Amines*.

[10] Cacabelos R. (1990). Histaminergic system: neuroendocrine function of brain histamine. *Methods and Findings in Experimental and Clinical Pharmacology*.

[11] Nautiyal K. M., Ribeiro A. C., Pfaff D. W., Silver R. (2008). Brain mast cells link the immune system to anxiety-like behavior. *Proceedings of the National Academy of Sciences of the United States of America*.

[12] Dong H., Zhang W., Zeng X. (2014). Histamine induces upregulated expression of histamine receptors and increases release of inflammatory mediators from microglia. *Molecular Neurobiology*.

[13] Iida T., Yoshikawa T., Matsuzawa T. (2015). Histamine H_3_ receptor in primary mouse microglia inhibits chemotaxis, phagocytosis, and cytokine secretion. *GLIA*.

[14] Zhu J., Qu C., Lu X., Zhang S. (2014). Activation of microglia by histamine and substance P. *Cellular Physiology and Biochemistry*.

[15] Afrin L. B., Pöhlau D., Raithel M. (2015). Mast cell activation disease: an underappreciated cause of neurologic and psychiatric symptoms and diseases. *Brain, Behavior, and Immunity*.

[16] Shan L., Bao A.-M., Swaab D. F. (2015). The human histaminergic system in neuropsychiatric disorders. *Trends in Neurosciences*.

[17] Kim Y. S., Kim Y.-B., Kim W. B. (2015). Histamine resets the circadian clock in the suprachiasmatic nucleus through the H1R-CaV1.3-RyR pathway in the mouse. *European Journal of Neuroscience*.

[18] Muindi F., Colas D., Ikeme J., Ruby N. F., Heller H. C. (2015). Loss of melanopsin photoreception and antagonism of the histamine H3 receptor by ciproxifan inhibit light-induced sleep in mice. *PLoS ONE*.

[19] Parmentier R., Zhao Y., Perier M. (2015). Role of histamine H1-receptor on behavioral states and wake maintenance during deficiency of a brain activating system: a study using a knockout mouse model. *Neuropharmacology*.

[20] Yu X., Ye Z., Houston C. M. (2015). Wakefulness is governed by GABA and histamine cotransmission. *Neuron*.

[21] Yu X., Zecharia A., Zhang Z. (2014). Circadian factor BMAL1 in histaminergic neurons regulates sleep architecture. *Current Biology*.

[22] Cacabelos R., Yamatodani A., Fukui H. (1986). Age- and sex-related changes in histamine and H-1 receptors in the central nervous system following a long-term castration. *Bulletin of the Japanese Neurochemical Society*.

[23] Cacabelos R., Yamatodani A., Fukui H. (1987). Time- and dose-dependent responses of brain histamine to intracerebroventricular and intraperitoneal administrations of growth hormone-releasing factor (GRF1-44). *The Tohoku Journal of Experimental Medicine*.

[24] Cacabelos R., Yamatodani A., Niigawa H., Hariguchi S., Nishimura T., Wada H. (1987). Histaminergic neuromodulation of the release of vasopressin. *Neuroendocrinology*.

[25] Cacabelos R., Alvarez X. A., Franco A., Fernández-Novoa L. (1993). Dose- and time-dependent effects of histamine on hypothalamic levels of interleukin-1*β* in rats. *Agents and Actions*.

[26] Fernández-Novoa L., Franco-Maside A., Álvarez X. A., Cacabelos R. (1995). Effects of histamine and *α*-fluoromethylhistidine on brain tumor necrosis factor levels in rats. *Inflammation Research*.

[27] Cacabelos R., Yamatodani A., Niigawa H. (1989). Brain histamine in Alzheimer's disease. *Methods and Findings in Experimental and Clinical Pharmacology*.

[28] Cacabelos R., Álvarez X. A., Fernandez-Novoa L. (1994). Brain interleukin-1*β* in Alzheimer's disease and vascular dementia. *Methods and Findings in Experimental and Clinical Pharmacology*.

[29] Cacabelos R., Franco-Maside A., Álvarez X. A. (1991). Interleukin-1 in Alzheimer's disease and multi-infarct dementia: neuropsychological correlations. *Methods and Findings in Experimental and Clinical Pharmacology*.

[30] Antón Alvarez X., Franco A., Fernández-Novoa L., Cacabelos R. (1996). Blood levels of histamine, IL-1*β*, and TNF-*α* in patients with mild to moderate Alzheimer disease. *Molecular and Chemical Neuropathology*.

[31] Cacabelos R., Álvarez X. A., Franco A. (1992). Therapeutic effects of CDP-choline in Alzheimer's disease and multi-infarct dementia: psychometric assessment and immune function. *Annals of Psychiatry*.

[32] Cacabelos R., Álvarez X. A., Franco-Maside A., Fernández-Novoa L., Caamaño J. (1993). Effect of CDP-choline on cognition and immune function in Alzheimer's disease and multi-infarct dementia. *Annals of the New York Academy of Sciences*.

[33] Fernandez-Novoa L., Alvarez X. A., Franco-Maside A., Caamano J., Cacabelos R. (1994). CDP-choline-induced blood histamine changes in Alzheimer's disease. *Methods and Findings in Experimental and Clinical Pharmacology*.

[34] Álvarez X., Franco A., Fernández-Novoa L., Cacabelos R. (1992). Effects of anapsos on behavior and brain cytokines in rats. *Annals of Psychiatry*.

[35] Alvarez A., Miguel-Hidalgo J. J., Fernandez-Novoa L., Diaz J., Sempere J. M., Cacabelos R. (1997). Anapsos: neuroimmunotrophic treatment in Alzheimer disease and neurodegenerative disorders. *CNS Drug Reviews*.

[36] Fernández-Novoa L., Álvarez X. A., Franco A., Caamaño J., Cacabelos R. (1993). Histamine-induced interleukin-1 changes in the rat hypothalamus. *Methods and Findings in Experimental and Clinical Pharmacology*.

[37] Maneiro E., Lombardi V. R. M., Lagares R., Cacabelos R. (1997). An experimental model to study the cytotoxic effects induced by *β*-amyloid, histamine, LPS and serum from Alzheimer patients on rat cultured endothelial cells. *Methods and Findings in Experimental and Clinical Pharmacology*.

[38] Lombardi V. R. M., García M., Cacabelos R. (1998). APOE-induced microglial activation: an *in vitro* assay to screen sera from Alzheimer's disease patients and novel therapeutic compounds. *Methods and Findings in Experimental and Clinical Pharmacology*.

[39] Lombardi V. R. M., García M., Cacabelos R. (1998). Microglial activation induced by factor(s) contained in sera from Alzheimer-related ApoE genotypes. *Journal of Neuroscience Research*.

[40] Cacabelos R. (2005). Molecular genetics of Alzheimer's disease and aging. *Methods and Findings in Experimental and Clinical Pharmacology*.

[41] Lombardi V. R. M., Fernández-Novoa L., Etcheverría I., Seoane S., Cacabelos R. (2004). Association between APOE *ε*4 allele and increased expression of CD95 on T cells from patients with Alzheimer's disease. *Methods and Findings in Experimental and Clinical Pharmacology*.

[42] Haig G. M., Pritchett Y., Meier A. (2014). A randomized study of H3 antagonist ABT-288 in mild-to-moderate Alzheimer's dementia. *Journal of Alzheimer's Disease*.

[43] Grove R. A., Harrington C. M., Mahler A. (2014). A randomized, double-blind, placebo-controlled, 16-week study of the H_3_ receptor antagonist, GSK239512 as a monotherapy in subjects with mild-to-moderate Alzheimer's disease. *Current Alzheimer Research*.

[44] Sriskantharajah S., Ley S. C. (2010). Turning off inflammation signaling. *Science*.

[45] Shembade N., Ma A., Harhaj E. W. (2010). Inhibition of nf-*κ*b signaling by a20 through disruption of ubiquitin enzyme complexes. *Science*.

[46] Zhang Q., Raoof M., Chen Y. (2010). Circulating mitochondrial DAMPs cause inflammatory responses to injury. *Nature*.

[47] Xu J., Chi F., Guo T. (2015). NOTCH reprograms mitochondrial metabolism for proinflammatory macrophage activation. *The Journal of Clinical Investigation*.

[48] Yin Y., Li X., Sha X. (2015). Early hyperlipidemia promotes endothelial activation via a caspase-1-sirtuin 1 pathway. *Arteriosclerosis, Thrombosis, and Vascular Biology*.

[49] Qiu L.-Q., Lai W. S., Bradbury A., Zeldin D. C., Blackshear P. J. (2015). Tristetraprolin (TTP) coordinately regulates primary and secondary cellular responses to proinflammatory stimuli. *Journal of Leukocyte Biology*.

[50] Naegelen I., Plançon S., Nicot N. (2015). An essential role of syntaxin 3 protein for granule exocytosis and secretion of il-1*α*, IL-1*β*, IL-12B, and CCL4 from differentiated HL-60 cells. *Journal of Leukocyte Biology*.

[51] Yang J. H., Kim K. M., Kim M. G. (2015). Role of sestrin2 in the regulation of proinflammatory signaling in macrophages. *Free Radical Biology & Medicine*.

